# Prevalence, Disparities, and Trends in Obesity and Severe Obesity Among Students in the Philadelphia, Pennsylvania, School District, 2006–2010

**DOI:** 10.5888/pcd9.120118

**Published:** 2012-09-06

**Authors:** Jessica M. Robbins, Giridhar Mallya, Marcia Polansky, Donald F. Schwarz

**Affiliations:** Author Affiliations: Giridhar Mallya, Donald F. Schwarz, Philadelphia Department of Public Health, Philadelphia, Pennsylvania; Marcia Polansky, Drexel University School of Public Health, Philadelphia, Pennsylvania.

## Abstract

**Introduction:**

Epidemic increases in obesity negatively affect the health of US children, individually and at the population level. Although surveillance of childhood obesity at the local level is challenging, height and weight data routinely collected by school districts are valuable and often underused public health resources.

**Methods:**

We analyzed data from the School District of Philadelphia for 4 school years (2006–2007 through 2009–2010) to assess the prevalence of and trends in obesity and severe obesity among public school children.

**Results:**

The prevalence of obesity decreased from 21.5% in 2006–2007 to 20.5% in 2009–2010, and the prevalence of severe obesity decreased from 8.5% to 7.9%. Both obesity and severe obesity were more common among students in grades 6 through 8 than among children in lower grades or among high school students. Hispanic boys and African American girls had the highest prevalence of obesity and severe obesity; Asian girls had much lower rates of obesity and severe obesity than any other group. Although obesity and severe obesity declined during the 4-year period in almost all demographic groups, the decreases were generally smaller in the groups with the highest prevalence, including high school students, Hispanic males, and African American females.

**Conclusion:**

Although these data suggest that the epidemic of childhood obesity may have begun to recede in Philadelphia, unacceptably high rates of obesity and severe obesity continue to threaten the health and futures of many school children.

## Introduction

Childhood obesity increased dramatically in the latter part of the 20th century, making it a leading public health issue (1). In 2009–2010, 18.2% of US children aged 6 through 19 years were obese, although national data suggest that the prevalence of childhood obesity is plateauing (2). Arkansas data support this trend, while recent New York City data indicate that childhood obesity is decreasing (3-7); however, the long-term trends and applicability to other parts of the United States are uncertain.

Recent efforts have been made to assess the epidemic increases in childhood obesity in the United States through public health surveillance methods. An important context in which such surveillance can take place is the school, because most children aged 5 through 18 years are enrolled in school and 90% of these students attend public schools (8). Many schools have nurses or other trained health personnel on site, and increasing numbers of school districts have mandated routine weight screening for students. The Centers for Disease Control and Prevention commissioned a report to provide guidance for school-based body mass index (BMI) measurement programs (9); Arkansas mandated school statewide BMI screening beginning in 2003, and as of 2009, a dozen other states had followed suit (9). Nonetheless, few communities have obtained and analyzed their data to track childhood obesity at the local level (10).

To identify the baseline prevalence and trends in childhood obesity locally, we analyzed height and weight data for students in kindergarten through grade 12 in the School District of Philadelphia. The school district includes approximately 300 public and charter schools, educating approximately 200,000 students. More than 80% of students are of racial/ethnic minorities, and more than 50% are eligible for free or reduced-price meals based on family income.  Our primary goals were to assess the prevalence of obesity and severe obesity among children in the School District of Philadelphia and to determine whether the prevalence of obesity and severe obesity changed between 2006–2007 and 2009–2010, the most recent school year for which data were available.

Dr. Sam Posner, editor in chief of *Preventing Chronic Disease (PCD)*, had the opportunity to speak with the authors of this week’s publications about their research on the country’s childhood obesity epidemic. Dr. Giridhar Mallya, director of policy and planning for the Philadelphia Department of Public Health, and Dr. James Marks, senior vice president and director of the Health Group at the Robert Wood Johnson Foundation, shared their insights on the obesity epidemic and described the importance of the research featured this week in *PCD*.

Listen to the podcast interview Audio/Video file [MP3 – 5.5MB]

A transcript of this interview is also available. [DOC – 25KB]

## Methods

School District of Philadelphia records cover all students enrolled in Philadelphia public schools at any point during the year, a number that varied from 189,913 in 2006–2007 to 177,499 in 2009–2010 (T. Williams, written communication, April 2012). Students who attended charter schools exclusively were not included. Analyses were limited to students aged 5 through 18 for whom valid height and weight measurements were recorded. These students represented an increasing percentage of the total school population over the 4 school years included, from 61.6% in 2006–2007 to 70.4% in 2009–2010. The demographics of the total school population are presented in the Appendix.

Students’ heights and weights were measured by school nurses. Pennsylvania mandates that all children in kindergarten through grade 12 have their height and weight measured annually by a nurse or teacher. The Pennsylvania Department of Health’s Division of Chronic Disease Intervention and Division of School Health have developed a manual to provide guidance to school districts and other educational entities on best practices in measuring and reporting these data (11). The measured heights and weights were entered into a secure school district database with the date of the examination. BMI measurements for girls whose records indicated a pregnancy were excluded from these analyses.

BMI was calculated as weight (kg)/height (m^2^) and compared with sex and age-specific norms from CDC growth charts (12) to determine BMI percentile. Obesity was defined as a BMI at or above the 95th percentile. Severe obesity was defined as a BMI of 35 or higher or 120% or greater of the threshold for obesity, based on the recommendation of Flegal et al (13). The validity of the 99th percentile estimates that have been commonly used to characterize severe obesity among children is questionable; these estimates do not match well to the empirical data on which the CDC growth charts were based (13). We conducted a secondary analysis of BMI values above the 99th percentile criterion to assess the sensitivity of the results to the differences in definition of severe obesity.

The demographic variables examined were sex; race/ethnicity; socioeconomic status, using eligibility for free or reduced-price school meals as a proxy; and grade. Race/ethnicity patterns were different for boys and girls and are therefore presented separately by sex. Data were weighted for nonresponse so that the measured population would more accurately represent the entire school population. Weights were calculated by grade and racial/ethnic group.

Although no intentional sampling was conducted, we conducted significance tests using SAS version 9.2 (SAS Institute, Inc, Cary, North Carolina) to assess the likelihood that changes over time represented chance findings. We assessed the significance of changes across time by testing a linear variable for school year in multivariable models that included a race/ethnicity–sex term, age in years, grade, and eligibility for free or reduced-price meals, adjusted for clustering within schools. Separate models were also conducted for each demographic group to assess time trends within groups.

To assess the possibility of bias associated with missing data, we identified a subsample of schools in which missing data were minimized (defined as at least 75% of all enrolled students having been measured during the school year), calculated the prevalence of obesity in this high-response sample, and compared the results with those of the total study population.

## Results

Valid height and weight measurements were obtained for 61.6% of all students in 2006–2007, 66.4% in 2007–2008, 69.1% in 2008–2009, and 70.4% in 2009–2010. These figures exclude girls whose records indicated a pregnancy during the school year (n = 1,134 during all 4 school years, 0.48% of all measured female students).

The demographics of the study population were similar to those of the entire student population, except that a larger proportion of the total population was in grades 9 through 12 (31.5% in 2009–2010, compared with 26.4% of the measured population) and a smaller proportion was in kindergarten through grade 5 (46.7% compared with 51.0%) (Table 1). The demographic characteristics of the population were generally similar for all 4 years. Boys comprised approximately 51% of the student population throughout the study period. African Americans were the largest racial/ethnic group (60.1% of all students and 59.3% of those measured in 2009–2010), followed by Hispanics (17.6% of all students, 16.7% of those measured), non-Hispanic whites (13.6% of all students, 14.2% of those measured), and Asians (6.3% of all students, 7.2% of those measured). The most notable change over time was the increase in the proportion of students who were eligible for free or reduced-price meals (from 48.9% to 57.4% among all students and from 50.8% to 59.5% among those measured), a change that occurred primarily between the 2007–2008 school year and the 2008–2009 school year.

**Table 1:** Demographic Characteristics^a^ of Students Aged 5 Through 18 Years Whose Weight Status Was Assessed, Philadelphia School District, 2006–2010

Characteristic	School Year, n (%)			
	2006–2007	2007–2008	2008–2009	2009–2010
**Total**	114,721 (100.0)	119,793 (100.0)	121,413 (100.0)	121,798 (100.0)
**Grade**				
K-5	57,222 (50.1)	59,568 (49.9)	60,342 (49.9)	61,940 (51.0)
6-8	28,724 (25.1)	28,331 (23.8)	26,825 (22.2)	27,436 (22.6)
9-12	28,322 (24.8)	31,404 (26.3)	33,856 (28.0)	31,984 (26.4)
**Sex**				
Male	58,679 (51.2)	61,152 (51.1)	61,940 (51.0)	62,223 (51.1)
Female	56,042 (48.8)	58,641 (49.0)	59,473 (49.0)	59,575 (48.9)
**Race/ethnicity**				
African American	68,310 (59.5)	71,629 (59.8)	72,911 (60.1)	72,182 (59.3)
Hispanic	19,485 (17.0)	20,305 (17.0)	20,514 (16.9)	20,375 (16.7)
Non-Hispanic white	18,111 (15.8)	18,084 (15.1)	17,554 (14.5)	17,330 (14.2)
Asian	7,263 (6.3)	7,849 (6.6)	8,105 (6.7)	8,774 (7.2)
Other	1,552 (1.4)	1,926 (1.6)	2,329 (1.9)	3,137 (2.6)
**Eligiblility for free/reduced-price meals**				
Eligible	58,296 (50.8)	59,542 (49.7)	71,184 (58.6)	72,484 (59.5)
Not eligible	56,425 (49.2)	60,251 (50.3)	50,229 (41.4)	49,314 (40.5)

Abbreviation: K, kindergarten.

^a^ Values may not sum to total due to missing data.

In 2009–2010, the prevalence of obesity among Philadelphia’s public school students was 20.5% (Table 2). The prevalence was higher among students in grades 6 through 8 (23.0%), compared with both kindergarten through fifth grade (19.1%) and high school students (20.8%). Among boys in 2009-2010, Hispanics had the highest prevalence of obesity (25.6%), followed by whites (20.7%); among girls in 2009-2010, African Americans had the highest prevalence (22.7%), followed by Hispanics (20.6%). The lowest prevalence by far—less than half that of any other group—was among Asian females, at 8.9% in 2009-2010.

**Table 2:** Obesity^a^ Prevalence, Students Aged 5 Through 18 Years, by Demographic Characteristics, Philadelphia School District, 2006–2010

Characteristic	School Year, %				*P* Value for Trend^b^	% Change 2006–2007 to 2009-2010
	2006–2007	2007–2008	2008–2009	2009–2010		
**Total**	21.5	21.3	20. 6	20.5	<.001	−4.8
**Grade**						
K-5	20.3	20.2	19.1	19.1	<.001	−6.0
6-8	24.1	23.2	22.6	23.0	<.01	−4.7
9-12	21.4	21.4	21.2	20.8	.43	−2.4
**Sex**						
Male	21.7	21.3	20.3	20.4	<.001	−6.1
Female	21.3	21.3	20.8	20.6	.04	−3.3
**Male race/ethnicity**						
African American	20.7	20.1	19.1	19.1	<.001	−7.6
Hispanic	26.2	26.1	24.7	25.6	.13	−2.5
Non-Hispanic white	22.2	21.7	21.1	20.7	.01	−6.8
Asian	19.9	18.9	18.1	18.1	.01	−8.8
Other	20.4	20.7	19.5	19.3	.37	−5.7
**Female race/ethnicity**						
African American	23.0	22.9	22.8	22.7	.60	−1.2
Hispanic	22.3	22.4	21.1	20.6	<.01	−7.4
Non-Hispanic white	17.5	17.7	17.1	17.3	.45	−0.8
Asian	9.4	9.1	9.2	8.9	.57	−5.0
Other	18.6	18.8	17.4	17.1	.35	−8.2
**Eligibility for free/reduced-price meals**						
Eligible	21.4	21.3	20.5	20.7	.01	−3.3
Not eligible	21.6	21.2	20.7	20.2	<.001	−6.7

Abbreviations: K, kindergarten.

^a^ Obesity was defined as a body mass index ≥95th percentile, according to Centers for Disease Control and Prevention growth charts (12). Data were weighted for nonresponse so that the measured population would more accurately represent the entire school population.

^b^ Calculated using Wald χ^2^ test. All tests were controlled for other variables shown in the table.

Obesity declined slightly from 21.5% in 2006–2007 to 20.5% in 2009–2010, representing a 4.8% decrease (Table 2). Most of the decrease was between 2006–2007 and 2008–2009, followed by a leveling off in 2009–2010. This pattern varied by demographic group. At the beginning of the period, the prevalence of obesity was slightly higher among boys than girls (21.7% vs 21.3%); in the most recent school year this was reversed (20.4% among boys vs 20.6% among girls). Among high school students, the small decline in obesity was not significant. Trends within racial/ethnic–sex groups were significant for African American, non-Hispanic white, and Asian boys and for Hispanic girls. None of the subgroup changes between 2008–2009 and 2009–2010 was significant.

The prevalence of severe obesity in 2009–2010 was 7.9% (Table 3). Patterns of disparities in severe obesity were similar to those for obesity. Severe obesity was most prevalent among students in grades 6 through 8 (9.1% in 2009-2010), and the prevalence of severe obesity exceeded 9% among Hispanic boys and African American girls in 2009-2010. Trends over time in severe obesity were similar overall to those for obesity (Table 3). For the overall population, severe obesity prevalence decreased from 8.5% to 7.9% from 2006–2007 to 2009–2010, representing a 7.7% decrease. The largest significant changes were seen among African American boys and Hispanic girls. Results using the 99th percentile of BMI as a threshold (not shown) showed similar patterns, with slightly lower overall prevalence and somewhat larger declines over time (from 6.3% in 2006–2007 to 5.6% in 2009–2010).

**Table 3:** Severe Obesity^a^ Prevalence, by Demographic Characteristics, Students Aged 5 Through 18 Years, Philadelphia School District, 2006–2010

Characteristic	School Year, %				*P* Value for Trend^b^	% Change 2006–2007 to 2009-2010
	2006–2007	2007–2008	2008–2009	2009–2010		
**Total**	8.5	8.4	7.9	7.9	<.001	−7.7
**Grade**						
K-5	7.3	7.2	6.7	6.6	<.001	−8.7
6-8	10.0	9.7	9.0	9.1	<.001	−9.1
9-12	9.2	9.2	8.9	8.7	.29	−4.4
**Sex**						
Male	8.8	8.5	7.8	7.8	<.001	−11.9
Female	8.2	8.3	8.0	7.9	.10	−2.9
**Male race/ethnicity**						
African American	8.8	8.4	7.7	7.6	<.001	−13.8
Hispanic	10.7	10.4	9.4	9.8	<.01	−8.4
Non-Hispanic white	8.0	8.1	7.7	7.6	.12	−6.0
Asian	5.6	5.2	4.9	5.2	.26	−7.2
Other	8. 8	7.4	6.8	6.3	.03	−28.0
**Female race/ethnicity**						
African American	9.2	9.5	9.2	9.2	.49	−0.1
Hispanic	8.3	8.1	7.7	7.5	.01	−10.2
Non-Hispanic white	6.1	5.8	6.2	6.2	.86	1.0
Asian	2.2	2.2	2.2	2.1	.61	−4.1
Other	6.1	6.7	5.4	5.3	.23	−11.7
**Eligibility for free/reduced-price meals**						
Eligible	8.6	8.6	8.0	8.1	<.01	−6.3
Not eligible	8.4	8.2	7.9	7.5	<.001	−10.2

Abbreviation: K, kindergarten.

^a^ Severe obesity was defined as a body mass index ≥35 kg/m^2^ or being ≥120% of the threshold for obesity, based on the recommendation of Flegal et al (13). Data were weighted for nonresponse so that the measured population would more accurately represent the entire school population.

^b^ Calculated using Wald χ^2^ test. All tests were controlled for other variables shown in the table.

Some groups that had shown declines in obesity, severe obesity, or both, including those with the highest prevalence of severe obesity, experienced small nonsignificant increases during the 2009–2010 school year. Most schools contributed at least 1 school year to the high-response subset with measurements for 75% or more of enrolled students. Analyses limited to this subset did not differ substantially from results for the complete data.

## Discussion

Analyses of annually collected height and weight data on more than 100,000 public school children in Philadelphia from 2006–2007 to 2009–2010 demonstrated that the prevalence of obesity may be decreasing in small but potentially meaningful ways. However, trends were not consistent across subgroups, and obesity remains alarmingly high, particularly among some racial and ethnic minorities. Severe obesity, which confers the greatest short- and long-term risks to physical and emotional health, affects nearly 1 in 12 children in Philadelphia.

Some of these findings are consistent with national statistics on child overweight and obesity from the National Health and Nutrition Examination Surveys (NHANES) (2), although national rates of obesity in 2009–2010 were lower than those found among Philadelphia public school children. The differences were greatest for non-Hispanic whites, among whom 17.2% of boys and 13.0% of girls aged 6 to 19 years were obese nationally, compared with 20.7% of boys and 17.3% of girls aged 5 through 18 years in the School District of Philadelphia. This finding may reflect differences in socioeconomic status between whites in Philadelphia public schools and the US population overall. African Americans in the School District of Philadelphia had lower rates of obesity than those in the national data (19.1% for boys and 22.7% for girls in Philadelphia vs 25.4% for boys and 26.1% for girls nationally). In both the NHANES and School District of Philadelphia data, African American girls had higher rates of obesity than non-Hispanic white girls. In the NHANES data, African American boys also had higher rates of obesity than non-Hispanic white boys, although the opposite was true among Philadelphia public school children. This may again reflect the lower socioeconomic status of whites in the Philadelphia public schools compared with those in the national NHANES sample.

The NHANES study did not find evidence of any noticeable trends in obesity prevalence from 2007–2008 through 2009–2010. The differences seen between the School District of Philadelphia and NHANES data may reflect the different age ranges (5 through 18 in Philadelphia vs 6 through 19 in NHANES), different periods (2006–2010 vs 2007–2010), differences in measurement and methods, or genuine differences between Philadelphia public school children and national averages. The small size of the NHANES study population, which yielded 80% power to detect changes in obesity prevalence of 5% or more (14), may have precluded detecting declines in obesity over this period.

Routinely collected BMI data for public school children have been published only for a few jurisdictions, including New York City and Arkansas. The prevalence, disparities, and trends in obesity among public school children in New York City were similar to those seen here, with generally consistent small declines in obesity, including an apparent leveling off in both cities in 2009–2010. Among students in kindergarten through eighth grade, the prevalence of obesity in Philadelphia public school children was between 0.2% and 0.7% lower than among those in New York City in the same period. In 2009–2010, the prevalence of obesity in these grades was 20.3% in Philadelphia and 21.0% in New York City (7).

The prevalence of obesity found in Philadelphia public school children is also similar to statewide findings for public school children in Arkansas. The prevalence in the 2009–2010 school year was 21% in Arkansas and 20% in Philadelphia (3). Trends were generally declining or flat during the last several years. Many of the specific racial/ethnic differences noted, such as the marked sex disparity among Asian students, are consistent (3-6). Arkansas has not, however, reported the recent decline in obesity among African Americans noted in both Philadelphia and New York City.

Children affected by severe obesity face even greater health risks than obese children. Among children examined in the NHANES survey, those who were above the 99th percentile of BMI had higher mean blood pressures and insulin levels, lower mean high-density lipoprotein (HDL) cholesterol levels, and higher prevalence of metabolic syndrome than those who had BMI percentiles in the 95th to 97th range, putting them at greater risk of cardiovascular disease (15). In the HEALTHY study, a survey of primarily low-income and minority sixth-graders, students with BMI at or above the 99th percentile had higher blood pressure and insulin levels, lower HDL cholesterol levels, and larger waist circumferences than those who were moderately obese (16). Psychosocial comorbidities are also more severe among children and youth with severe obesity (17). Although the prevalence and consequences of severe obesity in children have prompted widespread interest in various interventions, including bariatric surgery for adolescents (18) and child welfare involvement (19), trends in severe obesity have been less widely studied or reported than those for overweight and obesity. Madsen et al, using different definitions of severe obesity (BMI ≥97th or ≥99th percentile), reported differing trends among subgroups of California students (20). Some of the patterns they found, such as continuing increases in severe obesity among African American girls, are not consistent with those found from analysis of School District of Philadelphia data. This inconsistency could reflect regional differences; our secondary analysis indicated that using BMI at or above the 99th percentile to define severe obesity would not affect these trends.

Many of the strengths and limitations of these data originate from the routine screening assessments of student populations conducted by school nurses. The data represent large, unselected populations and were collected by experienced clinical professionals; conversely, they were not collected under rigorous protocols or with consistent equipment, nor were they validated. Research on the accuracy of measurements taken in schools suggests that measurements taken by school nurses are of reasonably high quality (21). The substantial proportions of students that were not measured, especially in the higher grades, leave potential for selection bias, although our secondary analyses suggest that such bias is limited. Children who did not attend public schools during the school year, including those who attended private, parochial, and charter schools, were not included, and obesity prevalence and trends could differ in these groups. The similarities seen in both trends over time and disparities between racial/ethnic–sex groups when comparing the Philadelphia data with data from New York City and Arkansas strengthen our confidence in their accuracy.

Although these data do not allow us to say what is responsible for the apparent reversal of the trend toward increasing childhood obesity, greater attention has been paid to improving school health environments both nationally and in the School District of Philadelphia. Since 1999, the EAT.RIGHT.NOW. Pennsylvania Nutrition Education TRACKS program has provided nutrition education to all students and parents who are eligible for SNAP (the federal Supplemental Nutrition Assistance Program) and is now in more than 270 district schools (T.E. Wolford, written communication, March 2012). In 2004, the district beverage policy mandated the removal of all sodas and sugar-sweetened drinks from vending machines, and in 2006 snack standards were developed for á la carte and vending items. In 2006, the Philadelphia School Reform Commission passed a comprehensive School Wellness Policy with provisions for competitive foods, physical activity, and nutrition education. Finally, from 2009–2010, School Food Services began offering “universal” or free breakfast to all students, discontinued the use of fryers, and switched from 2% to 1% low-fat milk. In 2010, the Philadelphia Department of Public Health (PDPH) launched the Get Healthy Philly (www.foodfitphilly.org) initiative to improve nutrition and physical activity through citywide policy and systems changes. PDPH has partnered with public and private sector organizations, including the School District of Philadelphia, to decrease the population-level burden of obesity and related diseases, particularly among children. Such comprehensive efforts may help accelerate the decreases in BMI found in the study reported here.

The inconsistency of findings between subgroups and the small increases in obesity, severe obesity or both in some groups in the most recent year of data indicate that it is not yet certain that the epidemic increases in child obesity are over. Continued surveillance is required to clarify whether we are seeing minor inconsistencies in a continuing crisis or a true change in the epidemic.

In either case, the prevalence of unhealthy weight remains unacceptably high among public school children in Philadelphia, and the evidence that some groups are facing exceptionally high health risks associated with obesity is sobering. When almost 9% of all teenage students are severely obese, identifying effective means of preventing obesity in our children, helping those already affected to attain a healthier weight, and preventing the serious chronic health problems associated with obesity remain urgent public health responsibilities.

## Appendix. Demographic Characteristics of All Students Aged 5 Through 18 Years, Philadelphia School District, 2006-2010^a^

**Table:**

Characteristic	School Year, n (%)			
	2006–2007	2007–2008	2008–2009	2009–2010
Total	186,224 (100.0)	180,479 (100.0)	175,632 (100.0)	172,975 (100.0)
Grade				
K-5	83,155 (44.7)	81,395 (45.1)	80,417 (45.8)	80,723 (46.7)
6-8	43,591 (23.4)	40,679 (22.5)	38,288 (21.8)	36,967 (21.4)
9-12	58,711 (31.5)	57,677 (32.0)	56,197 (32.0)	54,533 (31.5)
Ungraded	728 (0.4)	728 (0.4)	730 (0.4)	752 (0.4)
Age, y				
5	7,960 (4.3)	7,819 (4.3)	7,889 (4.5)	7,904 (4.6)
6-8	40,298 (21.6)	39,384 (21.8)	39,720 (22.6)	39,718 (23.0)
9-12	54,123 (29.1)	52,048 (28.8)	50,581 (28.8)	50,435 (29.2)
13-15	47,144 (25.3)	43,872 (24.3)	40,576 (23.1)	39,098 (22.6)
16-18	36,699 (19.7)	37,356 (20.7)	36,866 (21.0)	35,820 (20.7)
Sex				
Male	96,119 (51.6)	93,092 (51.6)	90,583 (51.6)	89,046 (51.5)
Female	90,105 (48.4)	87,387 (48.4)	85,049 (48.4)	83,929 (48.5)
Race/ethnicity				
African American	117,064 (62.9)	111,813 (62.0)	107,887 (61.4)	104,007 (60.1)
Hispanic	30,878 (16.6)	30,948 (17.1)	30,225 (17.2)	30,536 (17.7)
Non-Hispanic white	25,502 (13.7)	24,558 (13.6)	23,830 (13.6)	23,459 (13.6)
Asian	10,548 (5.7)	10,539 (5.8)	10,606 (6.0)	10,901 (6.3)
Other	2,232 (1.2)	2,621 (1.5)	3,084 (1.8)	4,072 (2.4)
Eligibility for free/reduced-price meals				
Eligible	91,016 (48.9)	86,666 (48.0)	99,829 (56.8)	99,333 (57.4)
Not eligible	95,208 (51.1)	93,813 (52.0)	75,803 (43.2)	73,642 (42.6)

Abbreviation: K, kindergarten.

^a^ Values may not sum to total due to missing data.
